# Supplementary material for: The Association Between Internet Addiction and Adolescents’ Mental Health: A Meta-Analytic Review
Source: Behav Sci (Basel). 2025 Jan 23;15(2):116. doi: 10.3390/bs15020116 (PMC11851916; doi:10.3390/bs15020116)
Supplement: Supplementary file 1 [file behavsci-15-00116-s001.zip › Supplementary Material 2. PRISMA 2020 Checklist for structured summaries.pdf]

Supplementary Material 2. PRISMA 2020 Checklist for structured summaries

Table S2. PRISMA 2020 Checklist for structured summaries (Page et al., 2021)

**Section/topic**	**Ítem number**	**Checklist item**
TITLE		
Títle	1	Identify the report or publication as a systematic review.
ANTECEDENTES		
Objetives	2	Provide an explicit statement of the main objectives or questions that the review addresses.
METHODS		
Eligibility criteria	3	Specify the inclusion and exclusion criteria for the review.
Information sources	4	Please specify the information sources (e.g. databases, registers) used to identify the studies and the date of the last search of each of these sources.
Risk of bias in individual studies	5	Please specify the methods used to assess the risk of bias of the individual included studies.
Summary of results	6	Please specify the methods used to present and synthesise the results.
RESULTS		
Included studies	7	Provide the total number of included studies and participants and summarize relevant study characteristics.
Summary of results	8	Present results for the primary outcomes, preferably indicating the number of included studies and participants in each. If a meta-analysis was performed, state the summary estimate and confidence or credibility interval. If comparing groups, describe the direction of the effect (e.g. which group was favoured).
DISCUSSION		
Limitations of the evidence	9	Provide a brief summary of the limitations of the evidence included in the review (e.g. risk of bias, inconsistency – heterogeneity – and imprecision).
Interpretation	10	Provides a general interpretation of the results and their important implications.
OTHERS		
Financing	11	Specify the main source of funding for the review.
Registration	12	Provide the name and registration number.

This checklist retains the same items that were included in the PRISMA statement for abstracts published in 2013, but has been revised to be worded in a manner consistent with the PRISMA 2020 statement. In addition, it includes a new item that recommends that authors specify the methods used to present and synthesize the results (item n.8 6).
