# Supplementary material for: The Association Between Internet Addiction and Adolescents’ Mental Health: A Meta-Analytic Review
Source: Behav Sci (Basel). 2025 Jan 23;15(2):116. doi: 10.3390/bs15020116 (PMC11851916; doi:10.3390/bs15020116)
Supplement: Supplementary file 1 [file behavsci-15-00116-s001.zip › Supplementary Material 1. PRISMA Checklist.pdf]

Supplementary Material 1. PRISMA Checklist

Table S1. PRISMA 2020 Checklist (Page et al., 2021)

**Section/topic**	**Ítem number**	**Checklist item**	**Location of the item in the publication**
TITLE			
Títle	1	Identify the publication as a systematic review.	pp.1
ABSTRACT			
Structured summary	2	See the checklist for structured summaries of the PRISMA 2020 statement.	Table S2 (see in the Supplementary Material)
INTRODUCTION			
Justification	3	Describe the justification for the review in the context of existing knowledge.	pp. 4-5
Objectives	4	Provide an explicit statement of the objectives or questions addressed by the review.	pp. 5
METHODS			
Eligibility criteria	5	Specify the inclusion and exclusion criteria for the review and how studies were grouped for synthesis.	pp. 6
Sources of information	6	Specify all databases, registries, websites, organisations, reference lists and other resources searched or consulted to identify the studies. Specify the date each resource was last searched or consulted.	pp. 7
Search strategy	7	Present the full search strategies for all databases, registers and websites, including any filters and limits used.	pp. 7
Study selection process	8	Specify the methods used to decide whether a study meets the review's inclusion criteria, including how many review authors screened each record and each retrieved publication, whether they worked independently and, if applicable, details of any automation tools used in the process.	pp. 7-8
Data extraction process	9	Indicate the methods used to extract data from reports or publications, including how many reviewers collected data from each publication, whether they worked	pp. 8-9

		independently, the processes for obtaining or confirming data by study investigators, and, if applicable, details of any automation tools used in the process.	
List of data	10a	List and define all outcomes for which data were sought. Specify whether all outcomes compatible with each outcome domain were sought (e.g. for all measurement scales, time points, analyses) and, if not, the methods used to decide which outcomes to collect.	pp. 9 and Table S3 (see in the Supplementary Material)
	10b	List and define all other variables for which data were sought (e.g., participant and intervention characteristics, funding sources). Describe any assumptions made about any missing or uncertain information.	pp. 9 and Table S3 (see in the Supplementary Material)
Risk of bias assessment of individual studies	11	Specify the methods used to assess the risk of bias of the included studies, including details of the tools used, how many review authors assessed each study and whether they worked independently and, if applicable, details of the automation tools used in the process.	pp. 8
Effect measurements	12	Specify, for each outcome, the measures of effect (e.g., risk ratio, mean difference) used in the synthesis or presentation of results.	pp. 9
Synthesis methods	13a	Describe the process used to decide which studies were eligible for each synthesis (e.g., by tabulating the characteristics of the intervention studies and comparing them to the intended groups for each synthesis (item number 5).	pp. 7-10
	13b	Describe any methods required to prepare data for presentation or synthesis, such as the handling of missing data in summary statistics or data conversions.	
	13c	Describe the methods used to tabulate or visually present the results of individual studies and their synthesis.	
	13d	Describe the methods used to synthesise the results and justify your choices. If a meta-analysis has been performed, describe the models, the methods used to identify the presence and extent of statistical heterogeneity, and the software used.	
	13e	Describe the methods used to explore possible causes of heterogeneity between study results (e.g. subgroup analysis, meta-regression).	

	13f	Describe the sensitivity analyses that have been performed to assess the robustness of the synthesis results.	
Assessment of publication bias	14	Describe the methods used to assess the risk of bias due to missing results in a synthesis (arising from publication biases).	pp. 10
Assessment of the certainty of evidence	15	Describe the methods used to assess the certainty (or confidence) in the body of evidence for each outcome.	pp. 10
RESULTS			
Selection of studies	16a	Describe the results of the search and screening processes, from the number of records identified in the search to the number of studies included in the review, ideally using a flow chart (Figure 1).	pp. 11 and Table 2
	16b	Cite studies that apparently met the inclusion criteria but were excluded and explain why they were excluded.	pp. 10-11
Characteristics of studies	17	Cite each included study and present its characteristics.	pp. 11-12 and Table 2
Risk of bias of individual studies	18	Present the risk of bias assessments for each of the included studies.	pp. 14-15
Results of individual studies	19	Present, for all outcomes and for each study: a) summary statistics for each group (if applicable) and b) the estimate of effect and its precision (e.g., credible or confidence interval), ideally using structured tables or graphs.	pp. 12-15
Results of the synthesis	20a	For each synthesis, briefly summarise the characteristics and risk of bias among the contributing studies.	14-15
	20b	Present the results of all statistical syntheses performed. If meta-analysis has been performed, present for each meta-analysis the summary estimator and its precision (e.g., credible or confidence interval) and measures of statistical heterogeneity. If groups are compared, describe the direction of effect.	pp.12-13
	20c	Present the results of all investigations on possible causes of heterogeneity between study results.	pp. 12-13
	20d	Present the results of all sensitivity analyses performed to assess the robustness of the synthesised results.	pp. 15
Publication biases	21	Present assessments of the risk of bias due to missing results (arising from publication biases) for each synthesis assessed.	pp.14-15
Certainty of evidence	22	Present assessments of the certainty (or confidence) in the body of evidence for each outcome assessed.	pp. 12-13

DISCUSSION			
Discussion	23a	Provide an overall interpretation of the results in the context of other evidence.	pp. 17-20
	23b	Argue the limitations of the evidence included in the review.	pp. 20-21
	23c	Argue the limitations of the review processes used.	pp. 20-21
	23d	Argue the implications of the findings for practice, policy and future research.	pp. 21
OTHER INFORMATION			
Registration and protocol	24a	Provide the registration information for the review, including name and registration number, or state that the review has not been registered.	
	24b	Indicate where the protocol can be accessed, or state that no protocol has been written.	
	24c	Describe and explain any amendments to the information provided in the registration or protocol.	Data availability
Funding	25	Describe the sources of financial or non-financial support for the review and the role of funders or sponsors in the review.	pp. 22
Conflict of interest	26	Declare any conflicts of interest of the review authors.	pp. 22
Availability of data, codes and other materials	27	Specify which of the following are publicly available and where they can be found: data extraction form templates, data extracted from included studies, data used for all analyses, analysis code, any other material used in the review.	pp. 21
