# Supplementary material for: The Association Between Internet Addiction and Adolescents’ Mental Health: A Meta-Analytic Review
Source: Behav Sci (Basel). 2025 Jan 23;15(2):116. doi: 10.3390/bs15020116 (PMC11851916; doi:10.3390/bs15020116)
Supplement: Supplementary file 1 [file behavsci-15-00116-s001.zip › Supplementary Material 3. Coding of variables for analysis in SPSS.pdf]

Supplementary Material 3. Coding of variables for analysis in SPSS

Table S3. Coding of variables for analysis in SPSS

**Item**	**Abbreviation**	**Description**	**Coding**
**Extrinsic Variables**			
01	Code	Code to identify the article	b
02	Authors	Citation of authors	c
03	N.Authors	Number of authors	b
04	YearPubli	Year of publication of the study	b
05	Punt_QualityArt	Final article quality score	b
06	Porcent_Quality Art	Final article quality percentage	b
**Substantive variables: subject and sample variables**			
07	NTotal	Number of persons in the sample analysed.	b
08	Pocent_Women	Percentage of women in final total sample (from which the results have been obtained)	b
09	Age_M	Mean age of the final total sample (from which the results have been obtained)	b
10	Age_SD	Standard deviation of the mean age of the final total sample (from which the results have been obtained)	b
11	Nationality	Nationality of the persons in the sample: 1.East Asia / 2. West Asia / 3. South Asia / 4. South America / 5. Europe / 6. Mix (Europe, North America or West Asia Middle East)	d
**Methodological variables: Design variables**			
12	Design	Design of the study proposed in the article: 1. Cross-sectional / 2. Longitudinal.	d
**Methodological variables: Variables related to Internet addiction**			
13	Descrip_IA	Term used in the article that refers to Internet addiction. 1. Internet Addiction (IA) / 2. Problematic Internet Use (PIU) / 3. Problematic Smartphone Use (PSU) / 4. Problematic Social Media Use (PSMU) / 5. Addiction to Social Networks and Internet	d.
14	Tool_IA	Tool used for the measurement of Internet Addiction 1. IAT / 2. APIUS / 3. Young and De Abreu's ten-item Internet addiction test / 4. YDQ / 5. SAS-SV / 6. Social Media Disorder Scale / 7. CIUS / 8. BFAS / 9.CIAS-R / 10. ERA-RSI / 11. PIUQ-SF-6 / 12. Formulario 10 items IA Diagnostic Questionnaire (basado en el IAT)	d.
**Methodological variables: Variables related to depression**			
15	Descrip_Dep	Term used in the article that refers to Depression 1. Depression	d.

16	Tool_Dep	Tool used for the measurement of Depression<br>1. CES-D / 2. PHQ-9 / 3. SDS / 4. DSRS / 5. CDI-S / 6. MDIC / 7. SDHS / 8. DASS-21/ 9. BDI	d.
**Methodological variables: Variables related to anxiety and stress.**			
17	Descrip_Anx	Term used in the article that refers Anxiety<br>1. Anxiety / 2. Generalised Anxiety / 3. Social Anxiety	d.
18	Tool_Anx	Tool used for the measurement of Anxiety<br>1. MDCI / 2. GAD-7 / 3. SAS / 4. STAI-6 / 5. DASS-21	d.
19	Descrip_Stress	Term used in the article that refers to Stress<br>1. Stress	d.
20	Tool_Stress	Tool used for the measurement of Stress<br>1. DASS-21	d.
**Methodological variables: Variables related to Suicidal behaviour (Suicidal behaviour or Self-harm).**			
21	Descrip_Suic	Term used in the article that refers Suicidal behaviour or Self-harm.<br>1. SSIB (suicide and self-injury) / 2. NSSI (non suicidal self injury) / 3. Suicidal ideation	d.
22	Tool_Suic	Tool used for the measurement of refers Suicidal behaviour or Self-harm.<br>1. HBICA / 2. DSHI-Y / 3. Scale of Suicidal Ideation / 4. Form of questions proposed by the authors	d.
**Methodological variables: Variables related to psychological Wellbeing**			
23	Descrip_PsyWellbeing	Term used in the article that refers Psychological Well-being<br>1. Psychological distress / 2. Mental Health / 3. Psychological Well-being / 4. Psychological complaints / 5. Subjective well-being / 6. Emotional well-being / 7. Behavioural and emotional problems	d.
24	Tool_PsyWellbeing	Tool used for the measurement of refers Psychological Well-being<br>1.DASS-21 / 2. BRUMS / 3. "mental" ranking de Cantril's ladder y HBSC Symptom Checklist. / 4. SVS / 5. GHQ-12 / 6. 9-item Index of Well-Being to assess adolescent subjective well-being / 7. Escala de Afecto Positivo y Negativo (PANAS) / 8. SDQ / 9. Formulario de los autores (Participants' subjective well-being)	d.
25	Descrip_SEsteem	Term used in the article that refers Self Esteem<br>1. Self Esteem	d.
26	Tool_ SEsteem	Tool used for the measurement of refers Self Esteem<br>1. RSES / 2. MDIC / 3. 10-item Rosenberg Self-Esteem Scale / 4. EAR /5. SSES	d.
27	Descrip_BodyIm	Term used in the article that refers Body Image<br>1. Body Image Dissatisfaction (BID) / 2. Body Satisfaction	d.
28	Tool_BodyIm	Tool used for the measurement of refers Body Image<br>1. BIDS / 2. EAC	d.
**Methodological variables: Variables related to Internalizing Problems.**			
29	Descrip_ProbInter	Term used in the article that refers Internalizing Problems<br>1. Emotional Problems	d.

30	Tool_ProbInter	Tool used for the measurement of refers Internalizing Problems<br>1. SDQ	d.
**Methodological variables: Variables related to Externalizing Problems**			
31	Descrip_ProbExter	Term used in the article that refers Externalizing Problems (general measure)<br>1. Externalizing Behavioural Problems / 2. Behavioural Problems	d.
32	Tool_ProbExter	Tool used for the measurement of refers Externalizing Problems (general measure)<br>1. YSR / 2. SDQ	d.
33	Descrip_Tobacoo	Term used in the article that refers Tobacco use<br>1. Tobacco use	d.
34	Tool_ Tobacoo	Tool used for the measurement of refers Tobacco use<br>1. Socio-demographic survey / 2. CCD	d.
35	Descrip_Alcohol	Term used in the article that refers Alcohol use<br>1. Alcohol use	d.
36	Tool_Alcohol	Tool used for the measurement of refers Alcohol use<br>1. Socio-demographic survey / 2. CCD	d.
37	Descrip_Agres	Term used in the article that refers Aggressiveness<br>1. Aggressiveness / 2. Aggressiveness personality	d.
38	Tool_Agres	Tool used for the measurement of refers Aggressiveness<br>1. AQ / 2. BWAQ / 3. PQR	d.
39	Descrip_Impuls	Term used in the article that refers Impulsiveness<br>1. Impulsiveness	d.
40	Tool_Impuls	Tool used for the measurement of refers Impulsiveness<br>1. BISS-11	d.
41	Descrip_ DelinqBehav	Term used in the article that refers Delinquent behaviour<br>1. Delinquent behaviour	d.
42	Tool_DelinqBehav	Tool used for the measurement of refers Delinquent behaviour<br>1. 12-item Problem Behavior Scale to assess delinquent behaviors among adolescents	d.
**Outcome variables**			
Relationship between Internet Addiction-Depression			
43	IA_DEP_e	Type of statistic used<br>1. Pearson Correlation (r) /2. Odds ratio (OR) / 3. Means and SD / 4. Beta (B)/ 5. Other (medians and quartiles 1 and 3)	d.
44	IA_DEP_r	Result of the relationship between the indicated variables provided by the study.	b.
Relationship between Internet Addiction-Anxiety			
45	IA_ANX_e	Type of statistic used<br>1. Pearson Correlation (r) /2. Odds ratio (OR) / 3. Means and SD / 4. Beta (B)/ 5. Other (medians and quartiles 1 and 3)	d.
46	IA_ANX_r	Result of the relationship between the indicated variables provided by the study.	b.
Relationship between Internet Addiction-Stress			
47	IA_STRESS_e	Type of statistic used	d.

48	IA_STRESS_r	1. Pearson Correlation (r) /2. Odds ratio (OR) / 3. Means and SD / 4. Beta (B)/ 5. Other (medians and quartiles 1 and 3)<br>Result of the relationship between the indicated variables provided by the study.	b.
Relationship between Internet Addiction-Suicidal Behaviour or Self-Harm			
49	IA_SUIC_e	Type of statistic used<br>1. Pearson Correlation (r) /2. Odds ratio (OR) / 3. Means and SD / 4. Beta (B)/ 5. Other (medians and quartiles 1 and 3)	d.
40	IA_SUIC_r	Result of the relationship between the indicated variables provided by the study.	b.
Relationship between Internet Addiction-Psychological Well-being			
51	IA_PSYWellbeing PSICO_e	Type of statistic used<br>1. Pearson Correlation (r) /2. Odds ratio (OR) / 3. Means and SD / 4. Beta (B)/ 5. Other (medians and quartiles 1 and 3)	d.
52	IA_PSYWellbeing _r	Result of the relationship between the indicated variables provided by the study.	b.
Relationship between Internet Addiction-Self Esteem			
53	IA_SESTEEM_e	Type of statistic used<br>1. Pearson Correlation (r) /2. Odds ratio (OR) / 3. Means and SD / 4. Beta (B)/ 5. Other (medians and quartiles 1 and 3)	d.
54	IA_SESTEEM_r	Result of the relationship between the indicated variables provided by the study.	b.
Relationship between Internet Addiction-Body Image			
55	IA_BODYIM_e	Type of statistic used<br>1. Pearson Correlation (r) /2. Odds ratio (OR) / 3. Means and SD / 4. Beta (B)/ 5. Other (medians and quartiles 1 and 3)	d.
56	IA_ BODYIM _r	Result of the relationship between the indicated variables provided by the study.	b.
Relationship between Internet Addiction-Internalizing Problems			
57	IA_ProbINTERTotal_e	Type of statistic used<br>1. Pearson Correlation (r) /2. Odds ratio (OR) / 3. Means and SD / 4. Beta (B)/ 5. Other (medians and quartiles 1 and 3)	d.
58	IA_ProbINTERTotal_r	Result of the relationship between the indicated variables provided by the study.	b.
Relationship between Internet Addiction-Externalizing Problems			
59	IA_ProbEXTERTotal_e	Type of statistic used<br>1. Pearson Correlation (r) /2. Odds ratio (OR) / 3. Means and SD / 4. Beta (B)/ 5. Other (medians and quartiles 1 and 3)	d.
60	IA_ProbEXTERTotal_r	Result of the relationship between the indicated variables provided by the study.	b.
Relationship between Internet Addiction-Tobacco use			
61	IA_TOBACCO_e	Type of statistic used<br>1. Pearson Correlation (r) /2. Odds ratio (OR) / 3. Means and SD / 4. Beta (B)/ 5. Other (medians and quartiles 1 and 3)	d.
62	IA_TOBACCO_r	Result of the relationship between the indicated variables provided by the study.	b.
Relationship between Internet Addiction-Alcohol use			
63	IA_ALCOHOL_e	Type of statistic used	d.

64	IA_ALCOHOL_r	1. Pearson Correlation (r) /2. Odds ratio (OR) / 3. Means and SD / 4. Beta (B)/ 5. Other (medians and quartiles 1 and 3) Result of the relationship between the indicated variables provided by the study.	b.
Relationship between Internet Addiction- Aggressiveness			
65	IA_AGRES_e	Type of statistic used 1. Pearson Correlation (r) /2. Odds ratio (OR) / 3. Means and SD / 4. Beta (B)/ 5. Other (medians and quartiles 1 and 3)	d.
66	IA_AGRES_r	Result of the relationship between the indicated variables provided by the study.	b.
Relationship between Internet Addiction- Impulsiveness			
67	IA_IMPULS_e	Type of statistic used 1. Pearson Correlation (r) /2. Odds ratio (OR) / 3. Means and SD / 4. Beta (B)/ 5. Other (medians and quartiles 1 and 3)	d.
68	IA_IMPULS_r	Result of the relationship between the indicated variables provided by the study.	b.
Relationship between Internet Addiction-Delinquent behaviour			
69	IA_DELINQBehav_e	Type of statistic used 1. Pearson Correlation (r) /2. Odds ratio (OR) / 3. Means and SD / 4. Beta (B)/ 5. Other (medians and quartiles 1 and 3)	d.
70	IA_DELINQBehav _e	Result of the relationship between the indicated variables provided by the study.	b.

Coding

a. 0: No / 1: Yes / 8: Do not proceed / 9: Do not provide that information.

b. Indicate numerical value (Number)

c. Indicate the concept (Text).

d. Follow detailed coding in description.
