# Supplementary material for: The Association Between Internet Addiction and Adolescents’ Mental Health: A Meta-Analytic Review
Source: Behav Sci (Basel). 2025 Jan 23;15(2):116. doi: 10.3390/bs15020116 (PMC11851916; doi:10.3390/bs15020116)
Supplement: Supplementary file 1 [file behavsci-15-00116-s001.zip › Supplementary Material 4. Articles quality assessment by JBI scale.pdf]

Supplementary Material 4. Articles quality assessment by JBI scale

Table S4. Articles quality assessment (n=33) by JBI scale

**Author (year)**	**1**	**2**	**3**	**4**	**5**	**6**	**7**	**8**	**Puntuation (%)**
Wang et al (2022)	Yes	Yes	Yes	Yes	Yes	Yes	Yes	Yes	6 (75%)
Yi & Li (2021)	No	Yes	Yes	Yes	Yes	Yes	Yes	Yes	7 (87%)
Kojima et al (2021)	No	Yes	Yes	Yes	Yes	Yes	Yes	Yes	7 (87%)
Chen et al (2020)	Yes	Yes	Yes	Yes	Yes	Yes	Yes	Yes	8 (100%)
Zhai et al (2020)	No	No	Yes	Yes	Yes	Yes	Yes	Yes	6 (75%)
Liu et al (2021)	No	Yes	Yes	Yes	Yes	Yes	Yes	Yes	7 (87%)
Cao et al (2021)	No	Yes	Yes	Yes	Yes	Yes	Yes	Yes	7 (87%)
Chi et al (2019)	No	Yes	Yes	Yes	Yes	Yes	Yes	Yes	7 (87%)
Obeid et al (2019)	Sí	Yes	Yes	Yes	Yes	Yes	Yes	Yes	8 (100%)
Li, X. et al (2019)	No	Yes	Yes	Yes	Yes	Yes	Yes	Yes	7 (87%)
Li, G. et al (2019)	No	Yes	Yes	Yes	Yes	Yes	Yes	Yes	7 (87%)
Arrivillaga et al (2022)	Yes	Yes	Yes	Yes	Yes	Yes	Yes	Yes	8 (100%)
Souza-Pereira et al (2020)	No	Yes	Yes	Yes	Yes	Yes	Yes	Yes	7 (87%)
Boer et al (2020)	Yes	No	Yes	No	Yes	Yes	Yes	Yes	6 (75%)
Khasmohammadi et al (2020)	No	Yes	Yes	Yes	Yes	Yes	Yes	Yes	7 (87%)
Yurdagül et al (2021)	No	Yes	Yes	Yes	Yes	Yes	Yes	Yes	7 (87%)
Liu et al (2023)	No	No	Yes	Yes	Yes	Yes	Yes	Yes	6 (75%)
Yang & Zhu (2023)	No	No	Yes	Yes	Yes	Yes	Yes	Yes	6 (75%)
Huang et al (2023)	No	Yes	Yes	Yes	Yes	Yes	Yes	Yes	7 (87%)
Gao et al (2022)	No	Yes	Yes	Yes	Yes	Yes	Yes	Yes	7 (87%)
Peng et al (2022)	Sí	Yes	Yes	Yes	Yes	Yes	Yes	Yes	8 (100%)
Hamdan et al (2022)	No	Yes	Yes	Yes	Yes	Yes	Yes	Yes	7 (87%)
Fujita et al (2022)	Yes	Yes	No	No	Yes	Yes	Yes	Yes	6 (75%)

Peng et al (2021a)	Yes	Yes	Yes	Yes	Yes	Yes	Yes	Yes	8 (100%)
Peng et al (2021b)	Yes	Yes	Yes	Yes	Yes	Yes	Yes	Yes	8 (100%)
Tamarit et al. (2021)	No	Yes	Yes	Yes	Yes	Yes	Yes	Yes	7 (87%)
Wang et al (2021)	No	Yes	Yes	Yes	Yes	Yes	Yes	Yes	7 (87%)
Andrade et al. (2021)	Yes	Yes	Yes	Yes	No	No	Yes	Yes	6 (75%)
Pontes & Macur (2021)	Yes	Yes	Yes	Yes	No	No	Yes	Yes	6 (75%)
Xu et al (2020)	Yes	Yes	Yes	Yes	Yes	Yes	Yes	Yes	8 (100%)
Kuang et al (2020)	Yes	Yes	Yes	Yes	Yes	Yes	Yes	Yes	8 (100%)
Huang et al (2020)	No	Yes	No	Yes	Yes	Yes	Yes	Yes	6 (75%)
Mathew & Krishnan (2020)	Yes	Yes	Yes	Yes	Yes	Yes	Yes	Yes	8 (100%)

1. Were the criteria for inclusion in the sample clearly defined?; 2. Were the study subjects and the setting described in detail?; 3. Was the exposure measured in a valid and reliable way?; 4. Were objective, standard criteria used for measurement of the condition?; 5. Were confounding factors identified?; 6. Were strategies to deal with confounding factors stated?; 7. Were the outcomes measured in a valid and reliable way?; 8. Was appropriate statistical analysis used?
